# Evidence-based early rehabilitation for children with cerebral palsy: co-development of a multifaceted knowledge translation strategy for rehabilitation professionals

**DOI:** 10.3389/fresc.2024.1413240

**Published:** 2024-08-07

**Authors:** Jessica H. Hanson, Annette Majnemer, Filomena Pietrangelo, Leigh Dickson, Keiko Shikako, Noémi Dahan-Oliel, Emma Steven, Georgia Iliopoulos, Tatiana Ogourtsova

**Affiliations:** ^1^ School of Physical and Occupational Therapy, Faculty of Medicine and Health Sciences, McGill University, Montreal, QC, Canada; ^2^ Montreal Children’s Hospital, Research Institute of the McGill University Health Center, Montreal, QC, Canada; ^3^ Centre for Interdisciplinary Research in Rehabilitation of Greater Montreal, Montreal, QC, Canada; ^4^ Research Center of the Jewish Rehabilitation Hospital, Centre de Santé et de Services Sociaux de Laval, Laval, QC, Canada; ^5^ Research Center of the Shriners Hospital for Children, Montreal, QC, Canada

**Keywords:** cerebral palsy, evidence-based practice, knowledge translation, rehabilitation, early interventions

## Abstract

**Background:**

Cerebral palsy (CP) is the most common childhood physical disability. Early and evidence-based rehabilitation is essential for improving functional outcomes in children with CP. However, rehabilitation professionals face barriers to adopting evidence-based practices (EBP)s. The objective of this project is to develop a knowledge translation (KT) strategy to support CP-EBP among pediatric rehabilitation professionals.

**Methods:**

We follow an integrated KT approach by collaborating with clinician- and patient-partners. Partners engaged in co-design through team meetings and content review via email. The KT strategy comprises two components: (1) An electronic (e)-KT toolkit was created from summarized evidence extracted from randomized clinical trials on early rehabilitation for children with CP, and (2) a multifaceted online KT training program developed with guidance from a scoping review exploring effective KT strategies.

**Results:**

The e-KT toolkit summarizes twenty-two early interventions for children with or at risk for CP aged 0–5 years. Each module features an introduction, resources, parent/family section, and clinician information, including outcomes, intervention effectiveness, and evidence level. The KT training program includes three 10–15 min video-based training modules, text summaries, quizzes, and case studies. Site champions, identified as qualified rehabilitation professionals, were onboarded to support the site implementation of the training program. A champion-training booklet and 1-hour session were designed to equip them with the necessary knowledge/resources.

**Conclusion:**

The tailored, multifaceted, and co-designed KT strategy aims to be implemented in pediatric rehabilitation sites to support professional's uptake of CP-EBPs. Lessons learned from its development, including the co-development process and multifaceted nature, hold potential for broader applications in rehabilitation.

## 1 Introduction

Cerebral palsy (CP) is the most common childhood physical disability with lifelong health impacts for the child and family (1, 2). The clinical signs of CP progressively emerge in infancy as motor development occurs (2). In the first years of life for a child with CP, aberrant neural circuitry develops in brain regions due to the disuse of affected limbs (3). This can ultimately compromise motor learning and performance. Extensive animal and human research demonstrates that when provided early in life, intensive interventions can significantly improve motor and cognitive outcomes for children with CP (3–6). Furthermore, enhancing the health and functioning of children with CP through timely and evidence-based rehabilitation intervention can effectively optimize societal and individual healthcare costs (7). Rehabilitation professionals (i.e., physiotherapists, speech-language pathologists, and occupational therapists) often intend to offer evidence-based practices (EBP)s for children with CP. However, clinical settings often pose numerous barriers that impact the ability to stay up to date with EBPs, resulting in outdated and possibly ineffective rehabilitation interventions implemented instead. Additionally, this may result in delays in the implementation of EBPs, missing a critical period of brain plasticity.

Knowledge translation (KT), as the Canadian Institutes of Health Research outlines, constitutes a dynamic and iterative process encompassing synthesis, dissemination, exchange, and the ethically sound application of knowledge (8). The primary goal of KT is to improve the effectiveness of health services and products by “translating knowledge” to interested parties (8). Effective KT strategies are pivotal in actualizing research findings into practical applications (9). Previous research has pinpointed effective KT initiatives for rehabilitation professionals, such as workshops, EBP leaders (i.e., knowledge brokers, champions) and online tools, that have supported the uptake of early and evidence-based interventions for children with CP (10, 11).

Numerous studies recognize that effective KT strategies should be adapted to the specific context and meticulously planned after comprehensively assessing barriers and facilitators (12, 13). However, Imms et al. have underscored the challenges in tailoring a KT strategy for CP rehabilitation at the organizational level due to barriers in organizational activities, such as the absence of leaders in EBPs and access to resources. Thus, open-access, online KT tools, as a component of a KT strategy, may support the uptake of EBPs by engaging a broader population of rehabilitation professionals and can then be adapted to specific clinical contexts (10). For instance, in a single-masked clustered randomized clinical trial (RCT) within a specific organization (14), a multifaceted tailored KT strategy utilizing knowledge brokers, an online evidence library, and workshops significantly improved rehabilitation professionals’ EBP for children with CP. However, the transferability of multifaceted KT strategies to different organizations often remains to be discovered due to organizational barriers and differences (15). Given the provincial structure of the Canadian healthcare system, KT initiatives should aim to be implemented across the different provincial health jurisdictions to maximize impact and health outcomes.

There is a growing demand for effective KT initiatives in Canada to bridge the gap between research and clinical practice and optimize early and evidence-based rehabilitation interventions for children with CP. To that effect, we aimed to co-design and launch a multifaceted KT strategy, including an (1) electronic(e)-KT toolkit and (2) KT training program. This brief report aims to describe our co-design journey.

## 2 Methods

### 2.1 Theoretical approach

**Figure 1:**
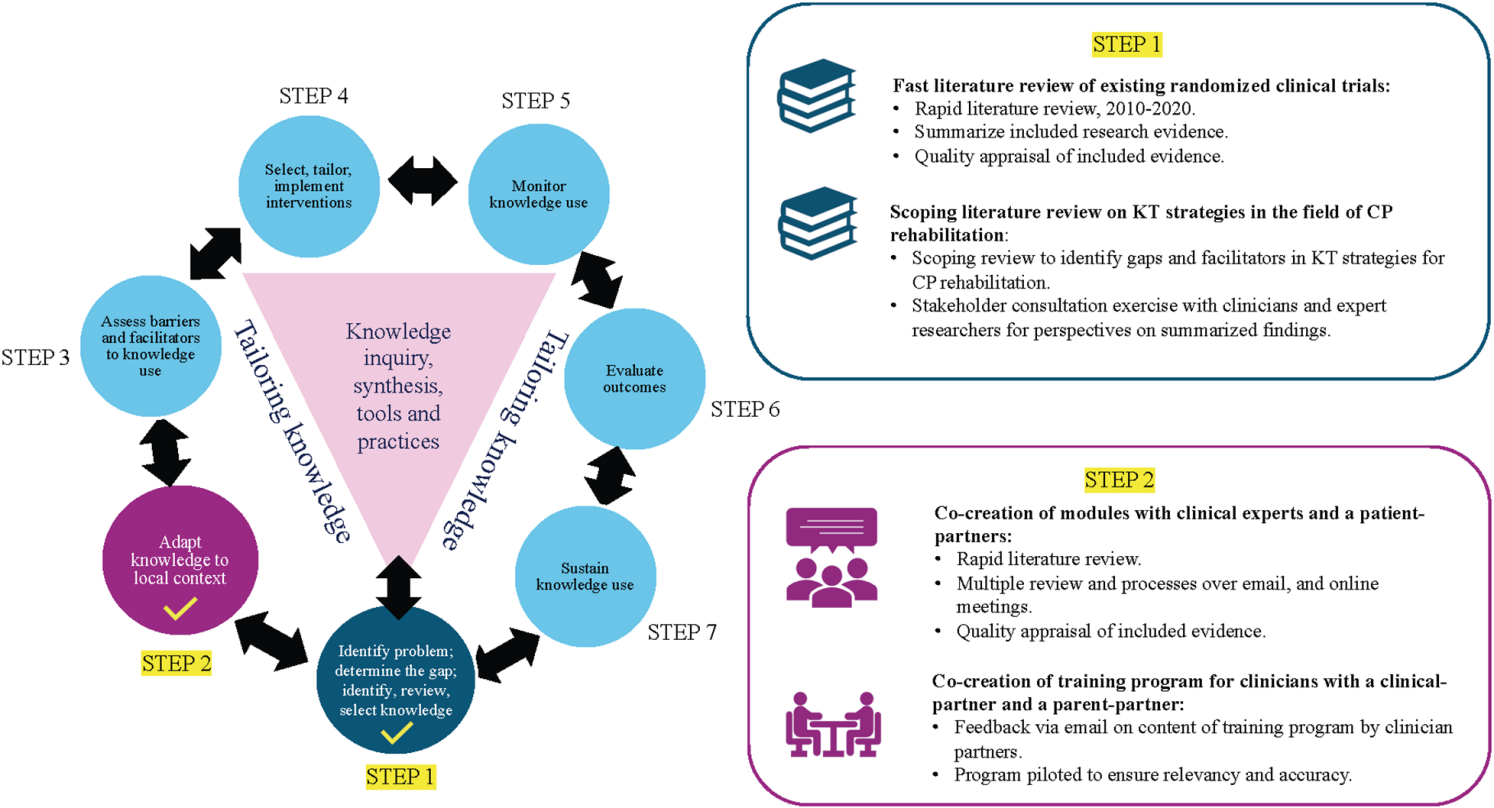
Knowledge-to-Action framework: *step 1* and *step 2*. KT, knowledge translation; CP, cerebral palsy. Figure adapted from (19), licensed under CC-BY-SA.

The development of the strategy is grounded in an integrated KT approach (iKT) (16), a robust method where knowledge-users are partners in the entire KT process (17, 18). The Knowledge to Action (KTA) framework (19) provides a structured process for moving through the KT process and was used to develop the multifaceted KT strategy. It includes a seven-step action cycle: (1) Identify the problem; determine the gap; identify, review, and select knowledge; (2) Adapt to local context; (3) Assess barriers/facilitators to knowledge use; (4) Select, tailor, implement interventions; (5) Monitor knowledge use; (6) Evaluate outcomes; and (7) Sustain knowledge use (19). As presented in Figure 1, to develop the e-KT toolkit and training program, we followed *Step (1) Identify the problem; determine the gap; identify, review,* and *select knowledge;* and *Step (2) Adapt to local context* (19).

### 2.2 Knowledge-user participation

Collaborations with five partners were established in our co-design process. Two were clinical experts: one occupational therapist with 11 years of experience in pediatric rehabilitation and one physical therapist with 13 years of clinical experience in pediatric rehabilitation. Two were patient partners: one mother of a 10-year-old boy with CP and one young adult with CP. One individual held the dual role of clinician- and patient-partner: occupational therapist with 5 years of clinical experience and a mother of a young child with developmental challenges undergoing diagnosis.

### 2.3 Study design and procedures

**Figure 2:**
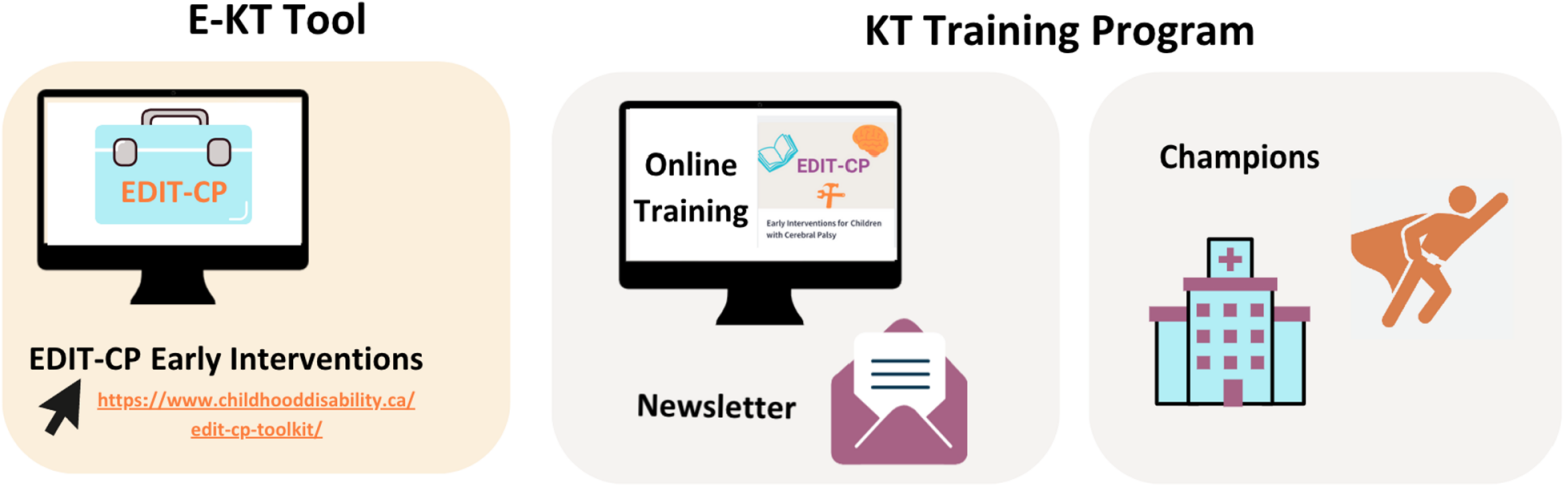
Diagram illustrating components of the *EDIT-CP: early intervention* multifaceted KT strategy. KT, knowledge translation; EDIT-CP, early detection and intervention toolkit for cerebral palsy.

The report discusses the development of the e-KT toolkit, Early Intervention and Detection Toolkit for Cerebral Palsy (EDIT-CP). The complete e-KT toolkit will be hosted on (https://www.childhooddisability.ca/edit-cp-toolkit/). The material available on the public site is subject to change based on this study and subsequent ones. This report focuses specifically on the EDIT-CP*: Early Intervention* e-KT toolkit. The EDIT-CP: *Early Detection* section for primary care physicians and families (https://www.childhooddisability.ca/early-detection-of-cp/) is described elsewhere (20). Secondarily, this paper focuses on the development of the EDIT-CP: *Early Intervention* KT training program. As shown in Figure 2, we present the KT strategy design in two parts: (1) e-KT toolkit and (2) KT training program.

#### 2.3.1 EDIT-CP: *early intervention* e-KT toolkit

The co-development of the e-KT toolkit includes *Step 1) Identify problem; determine the gap; identify, review, select knowledge* of the KTA framework. The toolkit includes a subsection for *rehabilitation professionals* (occupational therapists, physical therapists, and speech-language pathologists) and a separate subsection for *caregivers and families*.

The e-KT toolkit prototype was inspired by StrokEngine (www.strokengine.ca), an e-KT toolkit that provides evidence-based information about stroke rehabilitation and has been shown to be highly usable and navigable from rehabilitation professionals' perspectives (21). Additionally, patients/families reported being satisfied overall with the patient/family modules of StrokEngine and conveyed that the platform is a valuable resource (22). The senior author (TO) has developed over fifteen StrokEngine learning modules, including sections for rehabilitation professionals and for patients/families. The author applied this experience when designing the KT module prototypes for the e-KT toolkit.

The process was initiated with a rapid literature review of existing evidence-based CP rehabilitation approaches. RCTs published between 2010 and 2020, reflecting the latest developments in the field on rehabilitation interventions for young children (average less than seven) with or at risk for CP, were included in this process. Moreover, the references in published systematic reviews were examined to extract additional citations.

In *Step 2) Adapt to local context*, one clinical expert and one patient/clinician partner were trained in using the PEDro scale (23) for quality ratings to generate the level of evidence for every intervention included in the module. In addition, they extracted data in pre-developed extraction forms and drafted standardized studies’ summaries and conclusions. Based on the extracted data, one module per intervention was constructed (e.g., constraint-induced movement therapy). For the caregivers/family section, a prototype was first designed collaboratively by one parent/clinician partner and author (TO) for one intervention module (i.e., constraint-induced movement therapy) following a question-and-answer format, previously shown to be useful and understandable in the context of a KT toolkit in rehabilitation (22). This draft was sent to a second patient-partner (young adult with CP), and their feedback was requested on the content's completeness, appropriateness, and understandability. They were offered the option to provide comments/make edits directly on the created prototype using the Track Changes option in Microsoft Word. A period of two weeks was allotted to provide feedback.

#### 2.3.2 EDIT-CP: *early intervention* KT training program for rehabilitation professionals

In *Step 1) Identify problem; determine the gap; identify, review, select knowledge*, a scoping review was conducted by research team members (JH, AM and TO) exploring what KT strategies are used to promote evidence-based rehabilitation for children with CP and the impact of these strategies in promoting best practices (11). The Arksey & O’Malley framework (24), later expanded on by Levac et al. (25), was used to guide the review in six stages. The last stage included a consultation exercise where the summarized results were presented to rehabilitation professionals and researchers in this field for discussion and interpretation (25).

In *Step 2) Adapt to local context*, collaborative development activities with clinicians and patient-partners, including multiple review phases and online meetings, facilitated co-development. We developed a multifaceted KT training program using the e-KT toolkit to support rehabilitation therapists across Canada. The program includes a short online training course, weekly newsletters to prompt EBP, and support from EBP leaders. All course materials were translated from English to French and reviewed for accuracy by a qualified bilingual individual independent from the study.

**Figure 3:**
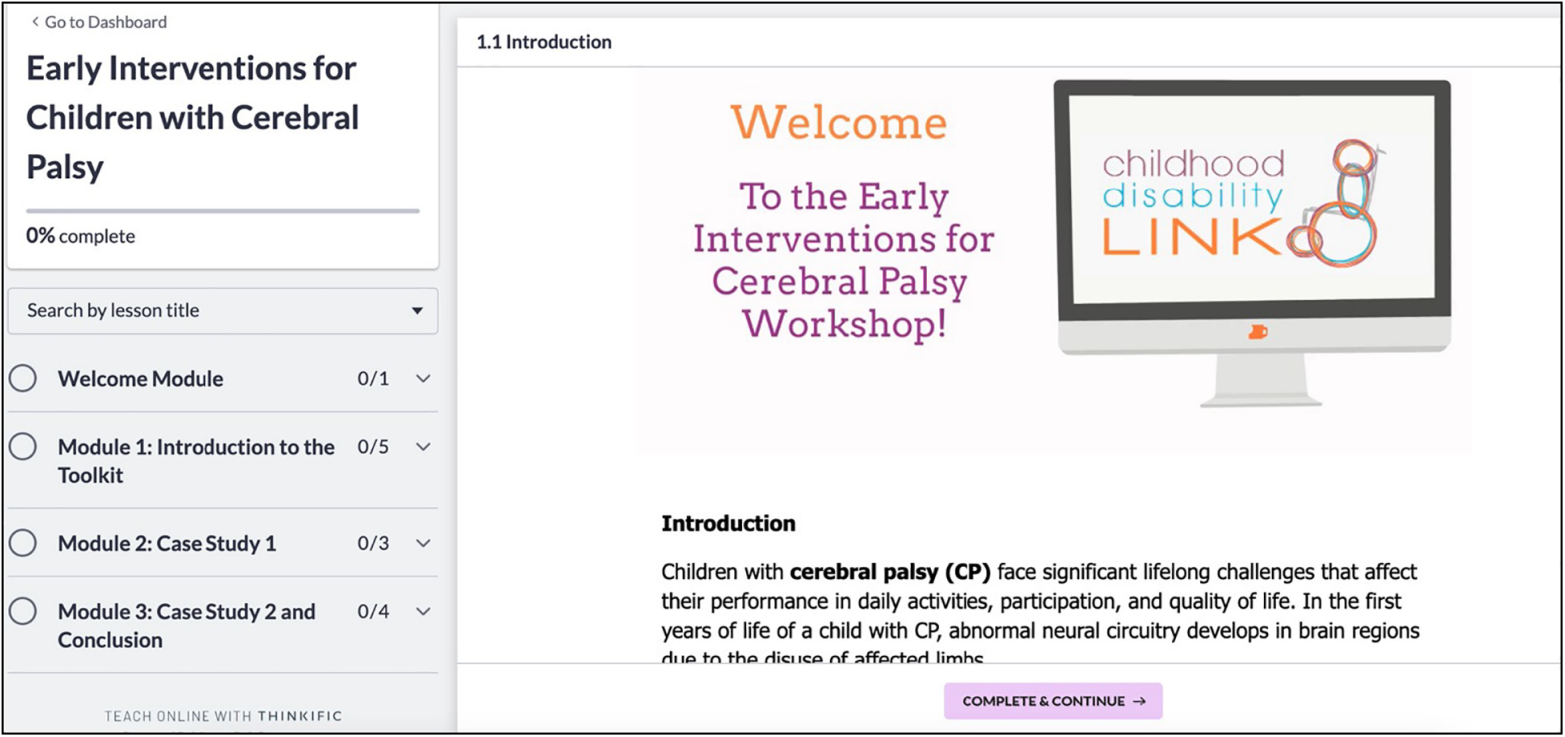
EDIT-CP online training course home page. EDIT-CP, early detection and intervention toolkit for cerebral palsy.

The online training course includes tutorial videos, quizzes, and a text summary. The training course was designed and hosted on Thinkific, a user-friendly and accessible course design platform (https://www.thinkific.com) (Figure 3). To create the videos, we used Animaker, a video and graphic design software (https://www.animaker.com). The training course content, including the videos, quizzes, and text summary, was first verified through direct feedback of content over email by researchers (*n* = 3) and clinician-partners (*n* = 2) to ensure suitability and acceptability. To access the course, interested parties must register via the platform's registration link, available at (https://earlyinterventionsforcp.thinkific.com). The registration process involves completing a form with basic information such as name and email address. Once registered, participants will receive instructions on how to access the training program materials. Please note that the availability of the course may change due to changes to funding and the learning module subscription. For questions and concerns regarding access to the training program or access to the French version, please email the corresponding author.

Based on our recent scoping review, reminders were shown to facilitate rehabilitation professionals' EBP uptake (11). Therefore, we designed a weekly newsletter to remind participating rehabilitation professionals about EBPs for children with CP and prompt them to use the e-KT toolkit to facilitate.

Moreover, to act as an EBP leader, we recruited a designated site champion for every site participating in the program. We recommend each site identify one champion, a qualified and accountable pediatric rehabilitation professional readily available to support local rehabilitation professionals' practical application of the e-KT toolkit and address any queries or concerns that may arise.

## 3 Results

### 3.1 EDIT-CP: *early intervention* e-KT toolkit

The e-KT toolkit is hosted on the Childhood Disability LINK website, a platform that provides up-to-date, reliable information about different types of childhood disabilities (https://www.childhooddisability.ca/edit-cp/). Twenty-two (*n* = 22) early interventions were identified across forty-one (*n* = 41) RCTs, and nineteen (*n* = 19) learning modules were developed (Supplementary Appendix 1). Children with different severities of CP are presented in the modules, with interventions targeting children with Gross Motor Function Classification System (GMFCS) Level I-III in 59% of the studied outcomes (Supplementary Appendix 2). Similarly, different types of CP are addressed in the early interventions, including spastic (in 20% of studied outcomes), ataxic (15%), dyskinetic (15%), mixed (10%), at risk for CP (14%) and diagnosed CP but type not specified (26%) (Supplementary Appendix 2). In total, one hundred-and-eight (*n* = 108) summaries were developed across the modules and included evidence on seventy (*n* = 70) different outcomes, with gross motor function, occupational performance, global development, dysphagia/feeding, and bimanual performance as the most common intervention targets (Supplementary Appendix 3). Regarding the quality of the included RCTs, 57.1% were high quality. Supplementary Appendix 4 presents the level and quality of evidence and common comparison interventions.

### 3.2 EDIT-CP*: early intervention* KT training program

The KT training program included an online course, site champions and a weekly newsletter (summarized in Table 1). The online course included three modules with approximately 10–15 minute (min) each of video content, followed by a short text summary of the video and a 5-question quiz. The course was estimated to be completed in 1 hour and 30 min. Informed by the scoping review findings, the course design followed a multifaceted, convenient, and integrated approach (11). Additionally, in accordance with a key finding from the scoping review, we included parent and clinician perspectives and input throughout the design and implementation of the training modules (11). Clinician-partners (*n* = 2) piloted the online course and felt the content was useful for practice. Specifically, feedback was given about Module 3.1, Case 2, and changes were made accordingly. One clinician-partner completed the course over a ‘lunch hour,’ suggesting it was easy and quick to complete.

**Table 1:** EDIT-CP KT training program.

Online course	
Module 1	Introduction to the toolkit
	• Section 1.1: *Introduction—*5* min video* ○ Overview of the online course and e-KT toolkit purpose. ○ Background on early interventions for young children with CP. ○ Importance of evidence-based interventions highlighted. ○ Testimonial video from a parent partner and a rehabilitation therapist partner.
	• Section 1.2: *Toolkit overview*—5* min video* ○ Detailing the e-KT toolkit design and content: including the introduction, resources, parent/family section and clinician information.
	• Section 1.3: *Quality/evidence rating*—5* min video* ○ Overview of methods of quality appraisal methods and evidence rating. ○ What ratings mean to support decision-making by rehabilitation professionals.
	• Text summary ○ Short summary of video content after each section.
	• Quiz 1 ○ 5 multiple choice questions. ○ Fixed questions related to the direct recall of the video content to ensure completion.
Module 2	Case study 1
	• Section 2.1: *Case study 1—10 min video* ○ 2-year-old boy with spastic unilateral CP, GMFCS Level III. ○ Overviews how therapists would use the e-KT toolkit for evidence-based intervention planning. ○ Interventions using e-KT toolkit for physiotherapists, speech-language pathologist and occupational therapists.
	• Text summary ○ Short summary of video content after each section.
	• Quiz 2 ○ 4 multiple choice questions. ○ Fixed questions related to the direct recall of the video content to ensure completion.
Module 3	Case study 2 and conclusion
	• Section 3.1: *Case study 2—* *8 min video* ○ 4-year-old girl with bilateral dyskinetic CP, GMFCS Level II. ○ Overviews how therapists would use the e-KT toolkit for evidence-based intervention planning. ○ Interventions using e-KT toolkit for physiotherapists, speech-language pathologist and occupational therapists.
	• Section 3.2: *Summary and conclusion—4 min video* ○ Overview of the course, learning goals, and future directions for using the e-KT toolkit.
	• Text summary ○ Short summary of video content after each section.
	• Quiz 3 ○ 5 multiple choice questions. ○ Fixed questions related to the direct recall of the video content to ensure completion.
Weekly reminders	
Newsletter	
	• Development of a weekly newsletter template to encourage continued use of the e-KT toolkit and completion of the training course via email. • Newsletter highlights new interventions added to the e-KT toolkit to keep rehabilitation professionals up to date.

Clinical champions	
Online training	
	• 1 h remote training sessions held with each champion by a research team member to summarize the training booklet and address questions or concerns.
Training booklet	
	• Developed to describe the e-KT toolkit contents, KT training program and being a site champion. • Contents include: ○ Introduction, Research Project Purpose, Research Project Methods, Role as Champion and Being a Champion.



KT, knowledge translation; CP, cerebral palsy; GMFCS, gross motor function classification system; EDIT-CP, early detection and intervention toolkit for cerebral palsy; min, minute; h, hour.

Module 1 of the online course includes three sub-sections. Section 1.1 contains a 5 min video presenting a brief overview of the online course and the purpose of the e-KT toolkit. The section consists of background knowledge and emphasizes the importance of early interventions for young children with CP. The section ends with a 1-minute testimonial video from one parent partner and one rehabilitation therapist partner. As highlighted by our scoping review, including testimonials about the importance of evidence-based interventions for CP could incentivize rehabilitation professionals to complete the short course and use EBP (11). Furthermore, Section 1.2 further details the e-KT toolkit, with a second 5 min video about the design of the toolkit and an overview of the e-KT toolkit content. Section 1.3 includes a 5 min video explaining quality appraisal methods and evidence rating. This section explores how evidence was integrated and appraised to support rehabilitation professionals’ decision-making and confidence in selecting appropriate evidence-based interventions from the toolkit. After each sub-section, there is a short text summary, and at the end of the module, a fixed quiz related to the direct recall of the content to ensure completion.

Module 2, Section 2.1 of the course presents a clinical case study of a child with CP. A 10 min video walks through the case and how therapists (physiotherapists, occupational therapists, and speech-language pathologists) would use the e-KT toolkit to support the development of an evidence-based intervention plan. The case was reviewed and verified by clinician-partners (one physical therapist and one occupational therapist) to ensure appropriateness and accuracy. A short text summary follows the video and a second quiz.

Module 3 of the course includes two sections. Section 3.1 presents a second clinical case study of a child with CP. This is a more complex case of a patient with a different CP type, severity and rehabilitation goals. An 8 min video discusses the case and how therapists would use the e-KT toolkit to support the development of an intervention plan. The case was reviewed and verified by clinician partners (one physical therapist and one occupational therapist) to ensure appropriateness and accuracy. A 4 min video in Section 3.2 provides an overview of the course, summarizing learning goals and future directions for using the e-KT toolkit in practice. A short text summary followed each section, followed by a final quiz.

A weekly newsletter template was developed to encourage participating rehabilitation professionals to continue using the e-KT toolkit to be sent via email. Furthermore, new interventions added to the e-KT toolkit are highlighted in the newsletter, keeping participants up-to-date and intrigued.

The findings of our scoping review supported the inclusion of site champions as EBP leaders in the KT training program to support rehabilitation therapists in using the e-KT toolkit in practice at their site (11). To train clinical champions, we created a training booklet describing the e-KT toolkit's contents (toolkit homepage, intervention homepage, and specific interventions). Additionally, the booklet included a summary of the multifaceted KT training program and its purpose. Information about each module was summarized. Furthermore, champions were asked to complete the KT training course. The training booklet articulates the role of the champion and the resources they would need to be a champion at their clinical site. A research team member (JH) met with each champion online for a 1 h training session, summarizing the training booklet and the e-KT toolkit and answering any questions the champions had.

## 4 Discussion

This report presents the development of a multifaceted KT strategy aimed at bridging the gap between research and clinical practice in early rehabilitation for children with CP. The co-creation of the KT strategy, together with rehabilitation professionals patient and parent partners, reflects a comprehensive approach grounded in an iKT framework (16). Moreover, engaging in a scoping review and consultation process yielded valuable insights into existing KT efforts in pediatric CP rehabilitation, informing a tailored and multifaceted strategy. The design of this KT strategy aimed to emphasize the importance of ensuring usability and relevancy for rehabilitation professionals and caregivers. Furthermore, it is recognized that it may not be as effective for researchers to take the lead in disseminating knowledge in a clinical setting, and it is important to work together with healthcare organizations and have an EBP leader support the KT process. Overall, we aimed to reduce barriers in our KT strategy by engaging in co-development with knowledge users, highlighting usability and feasibility in the design and encouraging the role of EBP site leaders.

The active involvement of key knowledge users, including clinician experts and patient partners, in our co-design process was foundational to developing the KT strategy. Following the iKT process allows for a more significant impact, often resulting in an end-product utilized by a greater audience and influencing the policy environment (26, 27). Involving knowledge-users in the KT development process ensures that the products developed reflect the unique perspectives, needs, and experiences of those living with CP and/or their caregivers (28). Additionally, including clinician-partner perspectives integrates real-world insights and practical considerations that may be overlooked, which was also essential for creating case study examples (29, 30). By engaging with those directly involved in the care and rehabilitation of children with CP, we expect to enhance the potential for successful implementation, ensuring the toolkit is not only evidence-based but also user-friendly, accessible, and feasible within the local context. Research has shown that customizing KT to a particular organization may improve clinicians’ acceptance and adherence (31, 32). Furthermore, working with knowledge users from the project's inception may lead to meaningful relationships and a sense of empowerment that can facilitate the research process (33). The iKT strategy integrates valuable perspectives and knowledge from parent and clinician partners by building respectful and honest relationships.

Following the KTA framework, *Step 1) Identify the problem; determine the gap; identify review,* we conducted a scoping review (11) prior to the final development of the KT training program. The scoping review explored current KT strategies for evidence-based pediatric rehabilitation for CP, aiming to identify the most effective approaches. This review also gave us insights into the barriers and facilitators faced by previous KT strategies (11). Further, the stakeholder consultation exercise added to the quality of our review and provided valuable perspectives and interpretations of the findings (25). The results revealed that adopting a tailored and multifaceted strategy and including an EBP leader or champion can enhance the implementation of KT (11). Conversely, the findings showed that a lack of protected time and funding was a common barrier to implementation efforts (11, 34). Therefore, convenience and accessibility are essential in KT strategies to promote uptake. The results emphasized that the successful implementation of a KT strategy was intricate and necessitated a comprehensive understanding of local barriers and contextual implementation (11). Overall, the review informed the development of the KT training program, ensuring it is relevant and follows best practices for KT interventions and tools. Previous research has shown that a scoping review is an effective way to understand the researcher's KT needs and ensure that stakeholder needs are identified in supporting best practices (35).

With insight from the scoping review and through the adoption of an iKT approach, we focused on the user-friendliness and relevancy of the KT strategy. Jargon is often a significant barrier to accessing and applying research evidence into practice, as it can be challenging for many non-academic readers to appraise scientific text and terminology (36). When developing the KT strategy, research evidence was summarized by the research team using lay language and reviewed by parents and clinician-partners to ensure understandability. It is shown that incorporating the end-user's personal preferences and needs in the KT strategy's design is beneficial for acceptance and uptake (37). Thus, in the e-KT toolkit, a section for families presents frequently asked questions and concerns that are more pertinent to families and may not be answered within the content meant for rehabilitation professionals. Additionally, as lack of time is a commonly identified barrier for EBPs (38), we aimed to create a convenient strategy with short videos and summarized research. Furthermore, including clinical case studies in the KT training program added a practical dimension, using clinical reasoning and illustrating the application of EDIT-CP interventions in context-based scenarios (39). Evidence suggests that designing effective and interactive content (i.e., quizzes, one-on-one support) to promote learner interaction is essential in maintaining their motivation for online learning (40). Thus, a straightforward, practical, and self-directed learning strategy targeted to meet the needs of rehabilitation professionals and families can contribute to maintaining participants’ adherence to the KT intervention.

By including site champions as EBP leaders in the KT training program, we aim to support the adoption of EBPs by participating rehabilitation professionals. The strategic inclusion of an EBP leader is meant to actively facilitate the adoption of EBPs by rehabilitation professionals in clinical settings (41). Additionally, EBP leaders can offer a tailored approach to KT strategies (i.e., applying the information to the local context), promoting ongoing feedback within programs and identifying areas requiring support (11). It is found that by including site champions to lead the KT strategy within their healthcare context, the motivation for rehabilitation professionals to participate in the KT strategy will likely gain further traction. Further, rehabilitation professionals may learn about tools at their site for mastering the EBP or approach, such as learning strategies or training that may help to administer the treatment approach to their patients. The correlation between sustained EBPs and effective leadership (41) suggests that KT strategies with EBP leadership are more likely to have a long-term impact that is pivotal in catalyzing change (42). To effect this change, EBP leaders must navigate contextually sensitive environments and negotiate timely and feasible responses to diverse knowledge user needs (42).

## 5 Limitations

Through developing the KT strategy, we aim to reduce challenges associated with the uptake of early and evidence-based interventions for children with CP. By engaging in a comprehensive iKT, incorporating the perspectives of clinician- and parent-partners, and leveraging the findings from a thorough scoping review, we have crafted a strategy to address the identified barriers. We recognize the level of engagement and satisfaction of knowledge users’ was not measured as a part of iKT. Hence, the absence of reliable and identifiable evaluation procedures for iKT can complicate the assessment of its influence on the research process or long-term results (43). Further, we recognize challenges regarding the sustainability of the toolkit and long-term funding, underscoring the complexity of implementing KT initiatives. Additionally, given that our strategy is aimed to be open access and designed to achieve scalability, there may be questions about the quality of the learning experience as smaller-scaled individualized learning strategies may be more effective (44). To mitigate these challenges, attention must be given to a comprehensive sustainability and dissemination plan to implement our KT strategy across health systems in Canada effectively. Future research efforts are needed to evaluate the iKT process and feasibility of the e-KT toolkit and training program to promote successful implementation at a larger scale. As we move forward, sustaining and funding KT initiatives emerges as a critical area requiring innovative solutions and continued advocacy for widescale dissemination.

## 6 Conclusion

We recommend the inclusion of the EDIT-CP*: Early Intervention* e-KT toolkit on healthcare organization websites, providing accessible resources for rehabilitation professionals and caregivers. Further, the EDIT-CP: *Early Intervention* KT training program should be a part of professional development curriculums, supporting knowledge about the importance of early EBPs for children with CP. In line with the next step of the KTA framework, *Step 3) Assess barriers to knowledge use*; a pre-post study is underway to evaluate the effectiveness, feasibility, and acceptability of the KT strategy in promoting EBP to support long-term implementation. The findings will inform modifications to the strategy; *Step 4) Select, tailor, and implement interventions.* Future directions of the project include *Step 5) Monitor knowledge* and *Step 6) Evaluate outcomes*, where strategic monitoring of knowledge utilization will be conducted, and evaluations will be carried out in collaboration with knowledge users. Lastly, *Step 7) Sustain knowledge use* is a continuous process where the research team updates the e-KT toolkit with emerging evidence.

By embracing a collaborative and evidence-driven approach grounded in iKT principles, this initiative endeavours to foster meaningful advancements in pediatric rehabilitation for children with CP. As we lay the foundation of a promising KT initiative, the lessons learned and the successes achieved offer a guide for rehabilitation professionals, researchers, and policymakers, ultimately supporting health outcomes for children living with CP and reducing the burden on the healthcare system.

## Data Availability

The raw data supporting the conclusions of this article will be made available by the authors, without undue reservation.
