# Supplementary material for: Evidence-based early rehabilitation for children with cerebral palsy: co-development of a multifaceted knowledge translation strategy for rehabilitation professionals
Source: Front Rehabil Sci. 2024 Aug 7;5:1413240. doi: 10.3389/fresc.2024.1413240 (PMC11335716; doi:10.3389/fresc.2024.1413240)
Supplement: Supplementary file 1 [file Datasheet1.docx]

**Supplementary 1**.

**Appendix 1.** EDIT-CP: Early intervention modules

| **EDIT-CP: Early Intervention Modules** |
| --- |
| Acupuncture |
| Auditory Tactile Vestibular Stimulation |
| Curriculum and Monitoring System |
| Constraint Induced Movement Therapy |
| Conductive Education |
| Coping and Caring for Infants with Special Needs |
| Deep Friction Massage |
| Electrical Stimulation |
| Goals Activity Motor Enrichment |
| Hand Arm Bimanual Intensive Training |
| Hippotherapy |
| Neurodevelopmental Treatment |
| Neurofacilitation of Developmental Reaction Approach |
| Nutrition and Feeding Rehabilitation   - *Functional Chewing Training* - *Nutrition Education Program* - *Oral Motor Therapy* - *Sensory Level Electrical Stimulation* |
| Occupational Therapy |
| Qigong Massage |
| Small Step Training |
| Treadmill Training |
| Vojta Approach |

**Appendix 2.** Severity and type of cerebral palsy targeted in reported outcomes

Cerebral palsy (CP); Gross Motor Function Classification System (GMFCS); Not applicable (NA).

**Appendix 3.**  The most common intervention targets

**Appendix 4**. Level and quality of evidence and type of comparison interventions


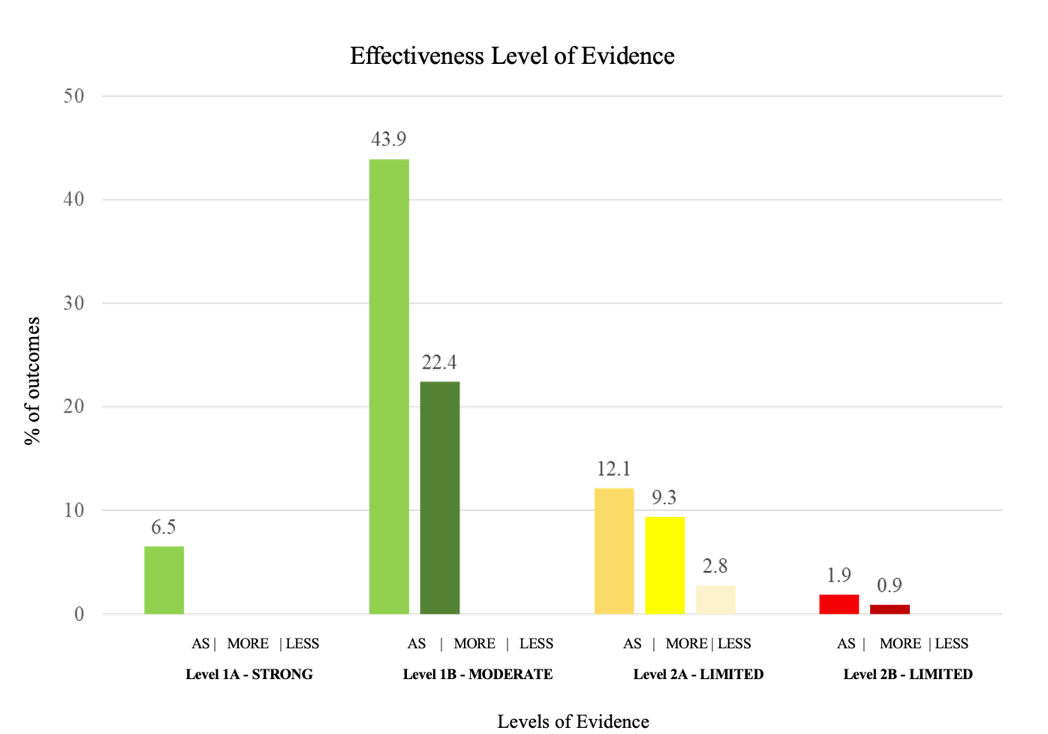


High, fair, and poor study quality refer to PEDro scores of ≤ 6/10, 4-5/10, and <4/10, respectively; Effectiveness of interventions shown as follows: as effective as the comparison intervention, more effective than the comparison intervention, and less effective than comparison intervention.
